# What Influences Urban Mothers’ Decisions on What to Feed Their Children Aged Under Five—The Case of Addis Ababa, Ethiopia

**DOI:** 10.3390/nu10091142

**Published:** 2018-08-22

**Authors:** Hanna Y. Berhane, Eva-Charlotte Ekström, Magnus Jirström, Yemane Berhane, Christopher Turner, Beatrix W. Alsanius, Jill Trenholm

**Affiliations:** 1 Department of Women’s and Children Health, International Maternal and Child Heath, Uppsala University, 751 85 Uppsala, Sweden; Lotta.Ekstrom@kbh.uu.se (E.-C.E.); jill.trenholm@kbh.uu.se (J.T.); 2 Addis Continental Institute of Public Health, 26751/1000 Addis Ababa, Ethiopia; yemaneberhane@gmail.com; 3 Department of Human Geography, Lund University, 223 62 Lund, Sweden; magnus.jirstrom@keg.lu.se (M.J.); christopher.turner@keg.lu.se (C.T.); 4 London School of Hygiene and Tropical Medicine, London WC1E 7HT, UK; 5 Department of Biosystems and Technology, Swedish University of Agricultural Sciences, 230 53 Alnarp, Sweden; Beatrix.Alsanius@slu.se

**Keywords:** urban mothers, child feeding/nutrition, qualitative methods, Ethiopia

## Abstract

Mothers carry the prime responsibility for childcare and feeding in low-income countries. Understanding their experiences in providing food for their children is paramount to informing efforts to improve the nutritional status of children. Such information is lacking in Sub-Saharan Africa. To understand what influences urban mothers’ food acquisition and their motivations for selecting food for their children, 36 in-depth interviews were carried out with mothers having children under five years of age. Interviews were conducted in the local language, audio-recorded, transcribed, and translated into English. Data were analyzed using thematic analysis which led to the identification of four major themes: mothers give-in to a child-driven diet; quick-fix versus the privilege of planning; keen awareness on food safety, nutrition, and diet diversity; and social, familial, and cultural influences. The findings indicate that child feeding practices are influenced by interlinked social and environmental factors. Hence, nutrition education campaigns should focus on targeting not only families but also their children. Attention should also be given to food safety regulations, as well as to the much-needed support of mothers who are struggling to ensure their children’s survival in low-income countries.

## 1. Introduction

In Sub-Saharan Africa, chronic childhood malnutrition is a major cause of child morbidity and mortality [1]. Though under-nutrition is still a major treat, the three simultaneous dimensions of malnutrition—undernourishment, micronutrient deficiencies, and over-nutrition—co-exist in developing countries [2]. Nutritional problems emanate from different underlying causes which include inadequate household food access and caregiver feeding practices [3,4]. Mothers, who are the primary care takers of children in this region, are responsible for purchasing, preparing, and household food allocation that affects the nutritional status of children.

Food-related decision making is a complex matter, and mothers need to consider the food supply, which is related to agricultural–food systems that affect the demand for and use of food [5]. Women’s decisions on what to feed their children are also influenced by price and environmental issues such as availability, type of outlets, food types, and location of shops, as well as social factors including knowledge of nutrition, social status, preference, peer pressure, time and effort required to prepare meals, prior bad or good food-related experiences, and cultural expectations [5,6,7,8,9]. These factors have a cumulative effect on the food purchasing practices of the mother, thereby determining what food is provided to the children. Available evidence highlights the importance of parents and caregivers as the primary influencers and gatekeepers of children's eating behaviors [10]. Therefore, understanding what influences mothers’ choices of food for their children is key to designing effective strategy to improve children’s diet and nutritional status. However, there is limited research on this issue in low-income countries. As urban settlements are increasing in Sub-Saharan Africa, food provision at a household level is becoming even more complex. Issues related to food systems in African cities have certain similarities [11] though much of them are not fully understood [12].

Due to urbanization, there has been a large shift in low-income countries from the traditional diet, rich in cereals and fiber towards more processed, calorie-dense foods that are rich in sugar and fat [13,14], in line with the global trends [15]. These rapidly growing food habit changes in low-income countries [16] are exacerbating the existing nutritional challenges [17]. In many urban areas, processed and ultra-processed food items are regarded as symbols of high economic status and are widely consumed [18]. A recent study in Ethiopia also showed that the consumption of saturated fats, sugary beverages, processed meats, cholesterol, and sodium is increasing in urban areas [19]. Considering that half of Africa’s population will be living in cities by 2050 [14], it is very important to understand the shifts in food habits and decisions that enforce the changes with further details to better inform policy and programs.

In cities that are rapidly growing, like our study area, people have limited access to cultivation land and are therefore highly reliant on purchased food [20], making the urban environment quite a different living space than the rural areas. The growing urbanization is disrupting the traditional eating/feeding practices, forcing households, particularly mothers, to navigate through the local market and make choices depending on their knowledge and economic status. Unfortunately, the food industry exploits the inherent human preference for energy-dense, smooth (refined, highly processed), salty, fatty, and sweet foods by making them widely available and affordable [21]—appealing for both mothers and their children. In financially constrained African cities, energy-dense but cheap nutrient-poor foods have become more accessible compared to healthy foods [6,22]. 

A combination of high rates of urban poverty, dependency of urban households on the market, and fluctuating food prices intensifies the food insecurity concerns among urban residents, leaving parents under the tremendous stress of making tough decisions on food choices. Caregivers, mostly mothers, who are responsible for providing to their children have to consider the options carefully before choosing what to buy. Having a more nuanced understanding of what influences mothers is key to designing effective interventions. Therefore, this paper explores urban mother’s food decision making through investigating her experiences within her food environment. 

### Conceptual Framework

This paper draws on the food environment conceptual framework developed by the Agriculture, Nutrition and Health Food Environment Working Group (ANH-FEWG) [23]. This framework draws its foundations from theoretical socio-ecological concepts and expands upon it to conceptualize the interaction with the wider food environment. The food environment according this framework has two domains that share an inter-related set of physical, economic, and socio-cultural dimensions. The external food environment domain consists of food availability, prices, vendor and product properties, marketing, and regulations, while the personal food environment domain encompasses accessibility, affordability, convenience and desirability at the individual level. In this study we focus on the personal domains and have used the definitions of Herforth and Ahmed [9] of the domains, which are more succinct and in line with our work. The availability and affordability domains focus on food availability in the market and the households’ ability to purchase food, respectively, while convenience considers how time and cost interrelate. When time is a scarce resource, ease of access and preparation may be even more important than the actual financial cost. Finally, desirability is seen as made up of internal factors (taste) and external factors (status of foods, cultural norms, advertising, product placement, and food quality). Though this framework served as a guide for organizing our results, this exploratory qualitative work concerning women’s decision of what to feed their children revealed more nuanced motivations which lie behind these very concrete factors. Turner et al. [23] in their review also point out the research gap in understanding the personal factors as well as the underutilization of qualitative studies. Hence, this study is sought to address this disconnect by focusing on individuals and understanding how they navigate within their wider food environment. 

## 2. Study Population and Methods

This study was conducted in Addis Ababa, Ethiopia, from March through November 2016. Addis Ababa is home to approximately 4 million people and is one of the most populous cities in Sub-Saharan Africa. Over the past two decades, the city has experienced a phenomenal economic, social, and spatial transformation which is expected to continue and is primarily attributed to the country’s double digit economic growth, rapid urbanization, and an expanding population [24]. Increased economic prosperity has translated into relatively more disposable income that can be used for purchasing foods, including those foods outside the traditional Ethiopian diet. In line with this, the retail sector in Addis Ababa is currently very diverse, ranging from the traditional open air market to modern supermarkets [24]. Despite the growing economy in the country, Addis Ababa is faced with poverty-aggravating factors such as massive rural–urban migration, poor housing, and unemployment, factors which counteract the progress of the city. Majority of the employed households (60%), have a monthly income of about US $68 and dependency ratio of 28% [25]. 

For this qualitative study, participants were sampled purposively with the help of the health extension workers who are employed by the government to provide primary health care at the neighborhood level. A maximum variation sampling technique was used to capture a wide range of perspectives related to child feeding by including mothers with children of a diverse age range (0–5 months, 6–23 months, and 24–59 months) to account for the different developmental stages of the child relative to their dietary needs [26]. Additionally, efforts were made to include mothers from various socio-economic levels in order to capture potential differences in their involvement in community work, educational status, work situation (working vs. stay at home moms), and number of children.

Members of the research team approached each woman identified for the study and explained the study objectives, confidentiality issues, data use and handling, and their right to withdraw from the study at any time without requiring a reason. After obtaining informed verbal consent from each participant, interviews were carried out in the privacy of their home or in other private locations where mothers felt comfortable to be interviewed. 

The dialogical approach as per naturalistic inquiry guided the interviews. This involves a more fluid conversation style of inquiry with open-ended questions versus a question–answer interview [27]. Initial questions were developed in consultation with the research team and were based on relevant literature. The questions included: “Can you tell us a little about your daily activities?”, “What types of food do you usually feed your child?”, “Are there any reasons why you give those food items to your child?”, “What are the popular foods given to children in your area?”, “What street foods are available in your area?”, and “Which street foods are commonly given to children?”. Some probes were also used to further facilitate dialogue, for example if a mother referred to her own different childhood foods, the questions “What is different from the way you were raised?” or “How do others raise their children with regards to child feeding?” were asked.

The first author observed several of the interviews and reviewed all field notes at the end of each day, discussing with the team how to fine-tune the interview process. Throughout the study, additional questions were added as new issues were raised, as is typical of emergent design in qualitative research. All interviews were carried out by experienced research assistants who had received training on interview techniques and the objectives of the study. 

A total of 36 interviews were completed; they ranged from 20 to 109 min in length. All interviews were tape-recorded, transcribed verbatim, and translated into English. Analysis was an ongoing process and formally commenced in the field, including fieldwork observations and daily recording of field notes. Thematic analysis was employed when all interviews were completed. To conceptualize what influenced mothers’ decisions in providing food for their children we used the lens of the personal food environment, which considers four dimensions: affordability, availability, convenience, and desirability [9,23]. Two of the authors then read and re-read the transcripts and identified themes independently. After the initial series of mapping, defining, and refining as per Braun and Clark [28], the dominant themes were discussed until consensus was attained among all the research team members. The themes were also presented in two seminars where feedback from other researchers promoted further clarity. 

### Ethical Considerations

Ethical approval for this study was obtained from the Institutional Review Board of Addis Continental Institute of Public Health. Before approaching individuals, the necessary permission was sought from the Lideta sub-city and all district (Woreda) health offices. All interviews were carried out in the privacy of the mothers’ homes; necessary precautions were taken to avoid intruders during the interview or avoid any situation that could make the mother uncomfortable. Data were then kept securely and were only accessible to the research team members.

## 3. Results

A systematic analysis of the qualitative data revealed four major themes: (1) Mothers give-in to a child-driven diet; (2) quick-fix solutions versus the privilege of planning; (3) keen awareness of food safety, nutrition, and diet diversity; (4) social, familial, and cultural influences. Each of the themes is elaborated below.

### 3.1. Mothers Give-in to a Child Driven Diet

For this theme, mothers expressed how they gave in to the child’s requests in many instances: they talked about giving food that the child preferred rather than what they thought was good for them, due to fear that the child may refuse to eat at all.

“I don’t know why he chose these foods (instant noodles). I cook very delicious foods but he refuses to eat them, especially lunch time. I don’t know why he only wants to eat instant noodles … So I often prepare that for him.”

Other mothers used food as a reward or an appeasement mechanism when the child was continuously crying, pleading, and nagging. In such instances, the food given appears to be less thought out and more impulsive. As this mother’s story conveys, the most accessible food at that moment, be it street food and/or sweets, was given to soothe the child:

“She mostly eats lollipops, whenever I go to work she starts crying … So I buy her a lollipop or biscuit to calm her down before I leave.”

Mothers also reported that what they pack in their child’s lunch box is highly influenced by fear that her child will feel inferior to other children and is driven by the child’s peer pressure. Mothers shared that they feel guilty when not being able to provide variety:

“I know kids love to have a variety of foods which are also good for their growth... But one may not afford to do that always … when they say ‘shiro (local stew made of chickpea or pea powder) again?’ (Denoting they have this inexpensive dish all the time) … you feel something inside and try to do something.”

Children hear and see different things at school and the neighborhood, and based on that they demand their mother to do something similar. In this example the child dictates what needs to be packed:

“Now that he sees different foods at school he says cook this and that … When I ask what to pack for school. He says: rice; macaroni, silsi (tomato sauce) with bread, egg sandwich.”

Some food retailors specifically target children, positioning their stores or vending stands alongside schools and thus tempting the children. When the children see those products, they start crying and pleading.

“There is ‘jelati’ (a kind of homemade popsicle) … chips, samosa, pasti (a kind of fried dough) ... there are many things sold especially at the doors of every school.”

This mother highlights, with the increasing street vendors around school, how difficult it is to adhere to the healthier home diet. Also, when the children see their friends having the street food, they want to act exactly like their friends.

“When he sees children eating chips bought from the street, he also wants us to buy it for him. I tell him it will make him sick … and I will make it for him at home but he doesn’t agree, he wants the one from the streets. The thing is you can’t escape … they are everywhere and continuously tempting him.”

### 3.2. Quick-Fix Versus the Privilege of Planning

Participants gave detailed accounts of the wide range of factors that influenced and constrained their decisions and choices in purchasing, cooking for and feeding their children. Mothers who were financially better off expressed a more planned style; they bought food items they needed in bulk, they opted for quality rather than quantity, and since they had space both for storage and cooking, they prepared most of their food at home.

“We buy teff (a type of grain, which is the staple for the country and used to make the flat bread called Injera) for a month … other ingredients like shiro (chickpea powder) and berbere (chili powder) for a year. Oil and the like we buy every three or four months.”

In contrast, other mothers said they were acutely aware that they were often unable to afford food and had to manage on a limited budget, devising different means to deal with this constraint. An approach that was mentioned is using sub-standard quality food to make sure the kids have something in their stomach:

“I sometimes even consider collecting spoiled fruits and vegetables from people’s house, even if I have to ‘pour sweats down my ass’ … I will do anything to bring home food for my kids.”

Another strategy followed by some mothers was to buy food from street venders that sell in small portions or to buy cheaper items as they encountered them rather than buying food in bulk and from their regular stores. Mothers also admitted to borrowing food items from their neighbors or taking store credits to cope with shortages as depicted in the example below:

“The most common thing we do is, if I am baking injera (local bread) my neighbor may come and take 1–2 injera, and I will do the same when I have shortage.”

Mothers in urban settings often express time constraint due to their engagement in economic activities or their social obligations. Thus, they sometimes tend to opt for quick-fix meals due to their convenience and ease of preparation and buying.

“Packed food like cerifam (brand name for one of the ready to make, packed baby food) … If I become very busy, I will give him by mixing it with boiled water, but I do this seldom.”

### 3.3. Keen Awareness on Food Safety, Nutrition, and Diet Diversity 

Almost all mothers who participated were well aware of the local nutritional recommendations; being particularly well informed about the importance of exclusive breast feeding, the correct timing to initiate complementary feeding, and the importance of balanced diet. They also agreed that nutrition knowledge influences their food choices:

“She (her baby) can’t have other food, she is too small for anything … she is just 45 days old. She will not start anything till she is 6 months. Even with my older child, he was entirely on breast milk until he was 6 months. It is after 6 months that I started food for him.”

Despite the fact that mothers were aware of the dietary recommendation, for the most part there appeared to be a ‘gap’ between nutritional awareness in theory and putting this knowledge into practice.

“I don’t think that chocolate is good; first it damages teeth; and for future as others said, it causes diabetic mellitus … But to avoid his disturbance, I buy it for him... regardless of the consequences.”

In some instances, despite the mother’s intention to provide diverse food for her family, due to her lack of in-depth knowledge on what counts as diverse, her efforts fell short of achieving what is best for the child. This mother indicated her idea of diversifying the food was to prepare stew with the same ingredient but changing the color and taste.

“If we prepare potatoes and carrots as a yellow sauce, tomorrow we will prepare potatoes with red sauce.”

Mothers demonstrated an awareness of food safety. Most of the mothers indicated that safety concerns are one of the things they consider in making decisions on the food products to give their children. They indicated that they are not happy when their children eat street food or outside the home as they fear contamination due to unhygienic food handling practices. Environmental contamination, especially of the street food, is another concern they voiced. Despite these foods being cheaper to buy, mothers revealed they preferred to prepare them at home instead.

“It’s cleanliness … it might not be that clean they might be careless about their utensils... too much dust blows on it … the cleanliness of the environment is worrisome.”

The other main concern was related to adulteration and fear of expired products. Mother mentioned they took extra care to select untampered products:

“I don’t buy packed juice … I don’t even like when adults drink it let alone children … because the expiry date may have passed … I heard traders import products passed their expiry date and sell it here by falsifying the expiry date (stamping altered dates) … So I don’t trust what is written on product.”

Mothers stated that they received dietary/food-related information from both informal and formal sources including schools, advertisements, and health workers (during the antenatal care visitisand from the health extension worker). The informal sources included: word of mouth, peer, rumors, and media.

“What I hear from media, from my friends, from my family about food and child feeding are important, I put in to practice what I hear from the different sources.”

The selection of food items for children was declared to also be influenced by the perceived health benefits or restrictions for different family members. If a family member has certain health condition that restricts him/her from eating certain food items, the family’s food habit will also change since most families eat together, and thus the children’s food consumption pattern could also be limited.

“… my husband suffers from ‘rhee’ (common local term used for gout) on his foot, therefore since it is not recommended for him to consume meat … the family also limits meat consumption to once a week.”

### 3.4. Social, Familial, and Cultural Influences

Mothers expressed that despite the cost of food, special holiday food was prioritized. Mothers indicated they would do everything in their power to make sure their kids got a good holiday meal. Animal source foods were identified by the mothers as hard to incorporate in a routine diet but formed part of holiday meals. As this expression from a mother captures:

“… animal source foods are not easy to find … we can’t afford eating eggs, meat or chicken regularly … those are special holiday meals for our family.”

The religious background of the family also influenced the type of food the children ate. As there are religious restrictions among both Muslim and Christian followers, there are certain foods that are not allowed to be consumed at all times and certain foods during fasting times.

“Eating ham is not allowed in our religion.”

“For breakfast we all eat together the same food, usually eggs or firfir (broken injera soaked in a sauce) except for Wednesdays and Fridays (which are fasting days) on which we don’t eat any dairy or any animal source food.”

The selection of food was also shown to be dictated by the perceived social norm. Mothers’ stated their desire to fit in and make sure their children identified with a given group and gained social approval.

“I usually give my children the same breakfast as the other kids in the neighborhood … think that is customary with families around. Bread with tea/milk is said to be good for children … especially milk since it has lots of nutrients.”

This mother’s expression also captures that social norms motivate the consumption of trendy foods which are more processed and not part of the staple diet:

“… There is much difference. Currently you prepare and give mashed meat like sandwich, macaroni, previously you won’t even get macaroni, let alone feed.”

Mothers also indicated that their familial roots had an influence on their food selection; in particular, some types of food were adapted from their ancestral roots. The intergenerational transmission of traditional foods is reflected by this mother’s expression:

“For example I am from Gurage ethnic group so sometimes I prepare kotcho (bread prepared from a false banana plant) with cottage cheese (ayibe) when it is not a fasting day... that is what I was used to in my upbringing.”

For most of the mothers, child-care and food preparation for the whole family was their responsibility, whether she was involved in additional income-generating activity or not. They considered all child-care responsibilities, from morning to evening, as the mothers’ responsibility, and fathers were only responsible for income-generating activities.

“I am responsible for all of the food purchasing and cooking … It is not convenient for him (referring to her husband) … He has to leave very early in the morning and comes back home quite late … his role is providing money.”

Though the gendered role of a mother as the preparer and feeder while fathers are the providers was typical for most women in our study; there were a few exceptions. For example:

“On his days off, for example on Sundays he helps out with everything starting from babysitting … we help each other.”

## 4. Discussion

This qualitative study found that mothers’ decisions on what to feed their children is influenced by a variety of factors including the children’s preference, their ability to plan for food, the information they have on nutrition,diet, and food safety and cultural/religious concerns, as well as changing societal and family values. These findings highlight the complexity involved in maternal decision making with regards to the food they gave their children.

The data in the study supports the importance/compatible use of the conceptual framework of the food environment as a basis for understanding and explaining how mothers make food choices for their young children. Within each theme, different dimensions of the external and personal food environment are reflected; in the first theme the influence of child preferences in maternal decisions was evident. This is in line with other studies whereby children’s taste, preference, and their hunger level [7] were identified to have an influence. Mothers in this study succumb to their children’s preferences not just because of the persistent pressure from the children but also due to added pressure from advertisements targeting children and mothers. Previous studies have shown that adverts have an effect on young children's subsequent attempts to influence shopping toward advertised foods which in turn drive their eating and/or snacking habits [29,30]. However, there are some child-oriented innovative initiatives in developed countries that encourage kids to make more nutritious food choices and become more physically active [31]. In this regard, social marketing campaigns in developing countries like Ethiopia are lagging behind commercial marketing in creating appeal for a more healthier diet alternatives. With what appears to be the child in the driver’s seat when making daily food choices, contextually suited innovative strategies are important to counteract the effects of advertisement and create a more discriminating audience that rejects processed foods and picks a healthy diet. A good example would be recreating/remarketing traditional foods into more peer-driven/modern versions, and school programs would be a natural choice for implementation.

It could also be argued that changes in family size and structure could be possible explanations why mothers give in, first of all since the birth rates in the study town (Addis Ababa, Ethiopia) are below replacement level [32]. Children are considered precious and privileged, and thus everything is done for their delight. Secondly, since mothers often work full-time they neither have the time nor the energy to cook, especially something their kids/family do not like to eat. This dimension of convenience has been supported by studies from Singapore and India: mothers were overwhelmed due to limited time availability to prepare, customize, and feed their children appropriately and adequately [33,34]. Considering the emphasis put on mothers as the ultimate purveyors of nutrition to their children, it is essential to support her in her endeavours and that includes supporting her health and wellbeing.

Affordability or financial/resource availability is the third dimension which was identified as one of the drivers for mothers’ decision in choosing food for their children, those who were better off had the opportunity to plan for a healthier diet while those with scarcity had to strategize to avoid hunger. Studies from high-income countries support that the better-off have a better opportunity for a healthy diet, since healthy items are more costly than refined, sugary, and fatty foods [35,36]. The participants in our study were very creative in strategizing the procurement and preparation of food. The various strategies used by families to cope with short- and long-term changes in household income and food insufficiency in this study have also been documented in other studies; measures taken include compromising quality and quantity (the frequency and amount) of food consumption [37,38]. Additionally, financial constraints also force mothers to opt for ready-to eat food like the instant noodles and to go for “modern” dishes like pasta, bread rather the traditional foods which require more resources to prepare. A review by Ruel and colleagues also highlighted that the food and fuel price increase during the 2007–2008 crisis forced households, especially for the urban poor, to increase consumption of street foods which can be a cheap source of energy and are also time savers [39]. The fear of incurring greater cooking fuel costs for the traditional preparation as opposed to the instant foods [40] contributes to the nutrition transition. 

Our participants were often knowledgeable about the child nutritional needs; however, we observed gaps between knowledge and practice: there was confusion about what constituted food diversity and they opted for giving sweets even though they know it is not good for the children. In a review, Worsley [41] argues that “nutrition knowledge” is a necessary but not sufficient factor for changes in consumers’ food behaviors.The behavior itself is influenced by a number of environmental and intra-individual factors, including motivations. In our previous work findings highlighted that mothers were functioning in an overwhelmed and thus compromised emotional state [42]. Though one would expect that that mothers’ emotional state would have a role in the interplay between motivational factors and information processing, studies weighing in on emotional aspects as a driver are lacking. Given that food choice and eating are already identified as highly complex and context-driven social practices [43] we need to consider additional dimensions like emotional status of the individual making these complex decisions in future studies. 

Food safety concern was one of the drivers of food choice. Food safety is a rising concern where poor agricultural, manufacturing, and hygiene practices contribute to a high rate of contamination with pesticide residues and heavy metal as well as microbial contamination [44,45]. Participants were almost unanimous in their declarations that all outside food is unhealthy. This was substantiated in a study from Ghana where respondents perceived food being offered in fast-food restaurants as unsafe and were thus concerned about several food safety issues [46]. Though food prepared in the house was perceived as healthy, studies are not widely available to determine whether traditional cooking techniques are indeed healthy. Herforth et al. [9] reflected that the term “desirability” as part of the food environment does not simply refer to preference, but rather to the external influence on preference like quality and sensory properties of food, knowledge, and norms of the consumers, in alignment with the findings of this study. 

Mothers’ food decisions are reinforced, driven by, or stem from traditions originating from religious, rituals, cultural taboos, or what they have learned from elders and their families. Furthermore, in this study fathers’ role was depicted as that of salaried work outside the house and as the main financial provider. There were some exceptions whereby fathers engaged and shared the responsibilities of child care and feeding, which may indicate traditional gender roles in transition. This finding is consistent with other findings from Ethiopia [47]. This study however focused on mothers, thereby curtailing deeper exploration into the role of fathers. Considering this as a limitation and the scarcity of literature on fathers and feeding, we would urge researchers to explore the role of fathers in child feeding.

The findings of this study indicate the importance of understanding the broad food environment which is crucial to the development of policy interventions aimed at improving the food supply as well as any nutirition-related goals. This is especially true for cities in low-income countries similar to our study setting which have experienced fundamental changes in the food systems [48] and are encountering emerging nutritional concerns of obesity and non-communicable diseases. However, there is lack of research focusing on food environment in low-income countries. This paper contributes to bridge that gap in information. By focusing on a rapidly changing urban area, this study highlights the complexity of the setting as well as the unique challenges that urban mothers face as they try to meet their responsibilities in feeding their children in a changing and complex food environment. This could serve as an vital input in the design of effective context-specific interventions. This qualitative work also fills the methodological gaps and serves as foundational knowledge for further qualitative exploration and quantitative studies. Quantifying the level of influence each of the different factors have on the food decision making would be an important adjunct to design effective interventions. 

### Reflexivity 

Though maximum efforts were made to minimize bias by training the data collectors to be sensitive, warm, and spend more time with the mother to create rapport, one of the limitations of this study is the possibility of social desirability bias as mothers would want to be perceived as “good mothers”. They may not always opt to tell us what they actually do but what they think researchers want to hear. Despite that, mothers appeared to appreciate a chance to express themselves and verbalized a host of challenges they deal with in feeding their offspring.

Throughout the research, issues related to trustworthiness were reflected upon by keeping an audit trail, having an iterative consulation with diverse research groups, and presenting results in different seminars. Mothers were selected from different districts and they represented diverse social environments. Additionally, Addis Ababa is one of the fastest growing cities in Africa, rendering these results as potentially transferable to other fast-growing cities on the continent, but consideration of how contexts differ remains at the discretion of the reader. In any case, it would be a good starting point for replication.

## 5. Conclusions

The findings of this study reveal a range of influences that mothers are faced with when making decisions on what to feed their children. Mothers living in rapidly urbanizing cities are pressured to make food choice decisions in contrary to their knowledge due to changes in the social practices, their roles at home, ever-present media advertisements, and rapid changes in the food environment. The extent to which each component influences mothers remains to be explored. Given that food choices and eating are highly complex and context-driven, future research should investigate the extent of these factors and how they interact to influence food choices made for children. For example, the extent to which financial constraints, cooking time and skills, infant acceptability, and food safety concerns contribute to the decision making process, particularly for lower-income parents, should be studied. Furthermore, the child-driven diet opens a whole range of possibilities for interventions that focus on the child as a purveyor of nutrition knowledge to the family versus the top-down approach. 
